# Supplementary figures and images for: CT584 Is Not a Protective Vaccine Antigen against Respiratory Chlamydial Challenge in Mice
Source: Vaccines (Basel). 2024 Oct 3;12(10):1134. doi: 10.3390/vaccines12101134 (PMC11512284; doi:10.3390/vaccines12101134)

Figure S1. Cm TC0873 and Ct CT584 are nearly identical

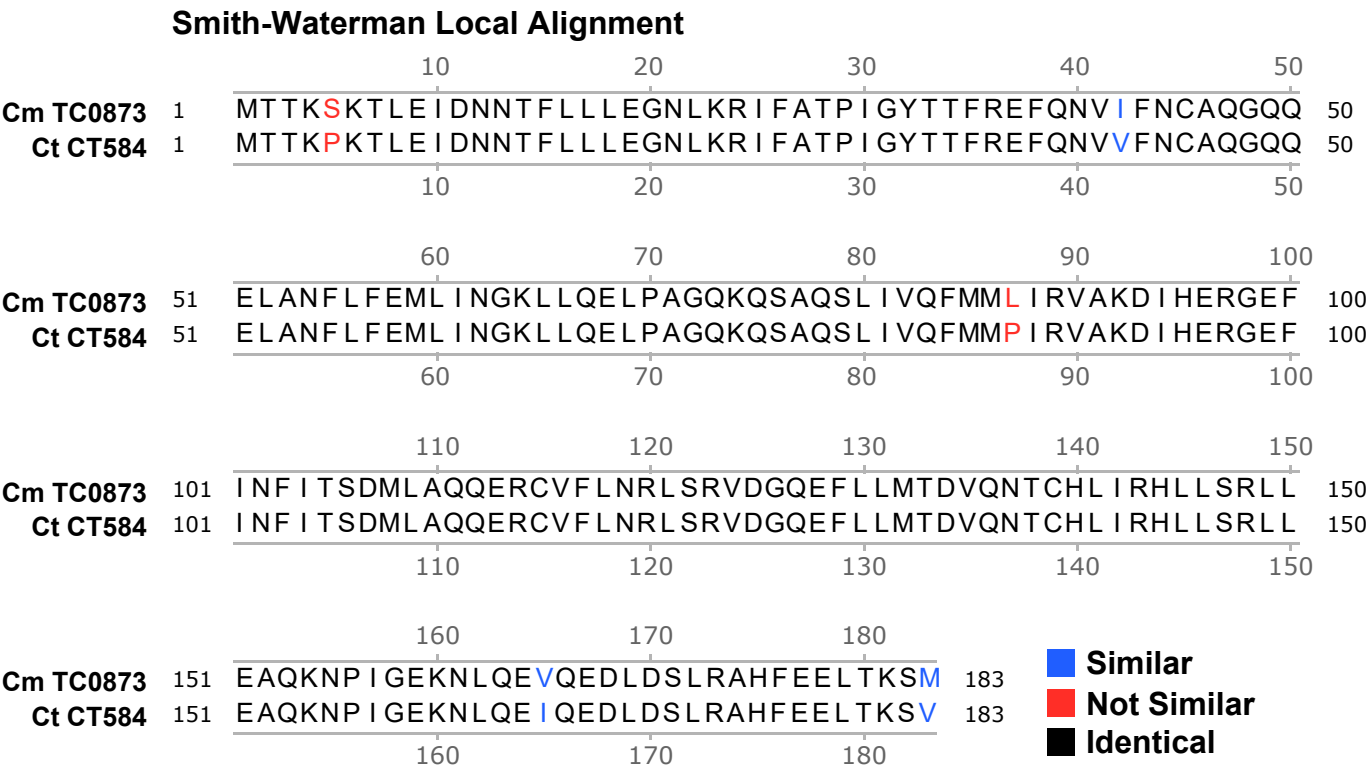

Supplement: Supplementary file 1 [file vaccines-12-01134-s001.zip › CT584 Figure S1.pdf]

Figure S2. E. coli DNA codon optimization for Ct CT584

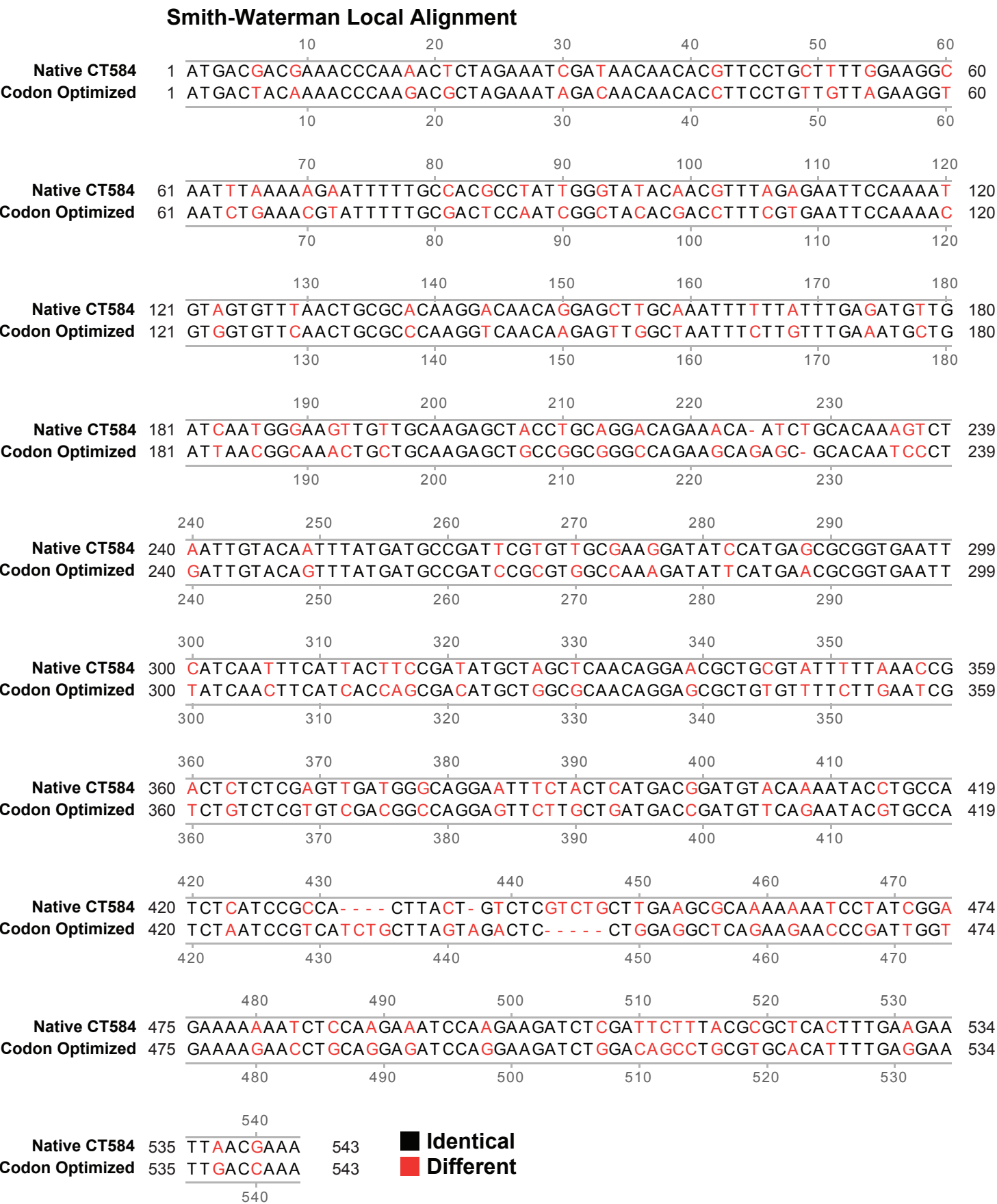

Supplement: Supplementary file 1 [file vaccines-12-01134-s001.zip › CT584 Figure S2.pdf]

Figure S3: Gating strategy for T-cell populations and Th1 cytokines

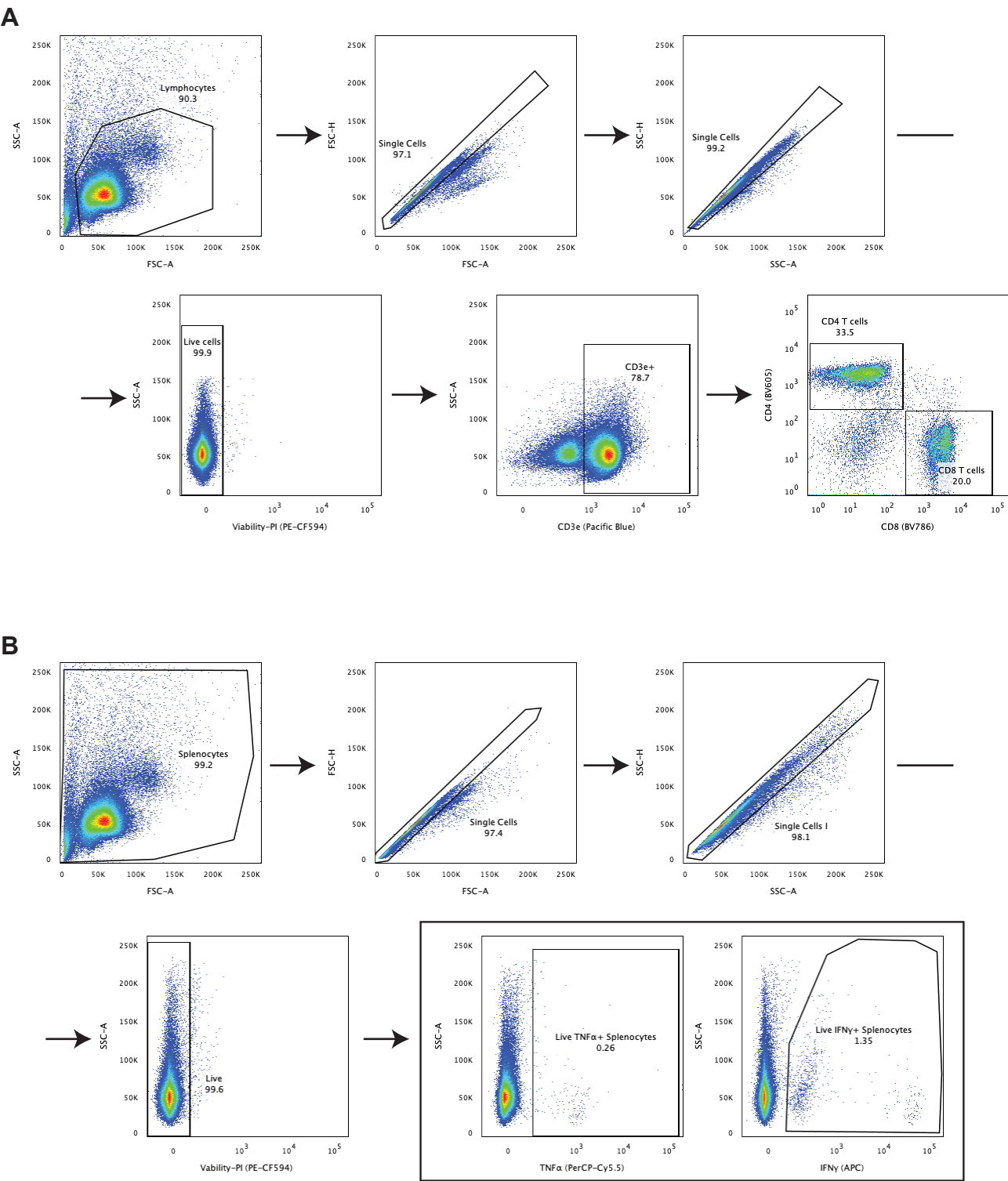

Supplement: Supplementary file 1 [file vaccines-12-01134-s001.zip › Figure S3.pdf]
